# New insights into recently emerged *Leucocytozoon caulleri* infection in Egyptian broiler flocks through clinical, pathological, hematological, and molecular investigation

**DOI:** 10.1038/s41598-025-26311-7

**Published:** 2025-12-03

**Authors:** Moustafa S. Abou El-Fetouh, Nahla AG. Ahmed Refat, Nora M. Elseddawy, Tarek Khamis, Mohamed A. A. Abdalla

**Affiliations:** 1 Department of Pathology, Faculty of Veterinary Medicine, Zagazig University, Zagazig, 44519 Egypt; 2 Department of Pharmacology, Faculty of Veterinary Medicine, Zagazig University, Zagazig, 44519 Egypt; 3 Laboratory of Biotechnology, Faculty of Veterinary Medicine, Zagazig University, Zagazig, 44519 Egypt

**Keywords:** Leucocytozoon caulleryi, Egypt, Broilers, Cell block, Histopathology, Real-time PCR, Zoology, Diseases

## Abstract

Leucocytozoonosis is a vector-borne blood protozoal disease that affects birds worldwide. Although Egypt has a large-scale poultry industry, data on *Leucocytozoon* infections in broilers remain limited. This study reports the first detection of leucocytozoonosis in 24 broiler farms (180,000 birds) in El-Sharkia governorate, Egypt. The affected broiler flocks were raised in open poultry farms located near agricultural rice fields and water sources during the late summer season, which are conditions favorable for vector proliferation. Clinically, infected birds exhibited depression, anorexia, weight loss, and anemia, with mortality rates ranging from 0.5% to 2%. Postmortem examination revealed widespread hemorrhages across multiple organs and a large pool of blood in the abdominal cavity. Microscopic examination of blood smears and cell block preparations showed the presence of various gametocytes within leukocytes and erythrocytes. Histopathological examination identified characteristic megaloschizonts in multiple organs, including the kidneys, liver, lungs, muscles, heart, intestine, pancreas, and spleen, accompanied by hemorrhages, necrosis, and mononuclear cell infiltration, primarily lymphocytes and macrophages. Molecular identification using real-time PCR confirmed *L. caulleryi* infection, corroborating the hematological and pathological findings. This study provides key insights into *L. caulleryi* diagnosis, promotes awareness among veterinarians and poultry producers, and highlights the need for improved biosecurity.

**Supplementary Information:**

The online version contains supplementary material available at 10.1038/s41598-025-26311-7.

## Introduction

Blood parasites of birds are classified into three genera: *Plasmodium*,* Haemoproteus*,* and Leucocytozoon*^1^. Leucocytozoonosis is a blood protozoal disease that affects many bird species and transmitted through insect bites. *Leucocytozoon* is a genus of parasites belonging to the family *Leucocytozoidae*, within the order *Achromatorida*, class *Aconoidasida*, subclass *Haemosporidiasina*, and phylum *Apicomplexa*. These parasites are intracellular and possess a specialized structure in their sporozoites^2^. There are about one hundred *Leucocytozoon* species that infect over 100 different species of birds. Many species are host-specific for either the intermediate or final host^3^. *Leucocytozoon caulleryi (L. caulleryi)* specifically infects domestic chickens^3^. The geographic distribution of the infection correlates with the presence of the intermediate host in the area. Infection primarily occurs in open poultry farms located near water sources and rice fields, which serve as natural habitats for the vector^4^. *L. caulleryi* exhibits a complex life cycle with two hosts involving birds and biting midges (*Culicoides*). Merogony, schizogony (tissue phase), and gametogony (blood phase) occur in the chickens, but sporogony takes place in the insect^1^. The biting midges inoculate the sporozoites into the chicken when taking blood meals. These sporozoites infect the liver and form primary schizonts, which mature and release primary merozoites. These merozoites invade the capillary endothelium of various organs and develop into megaloschizonts, which then release second-generation merozoites. These merozoites occupy erythrocytes and leukocytes to form gametocytes that mature and circulate in the blood. The biting midges suck the gametocytes, which fuse to form zygotes in the fly’s midgut, then develop into oocytes that contain motile sporozoites in the salivary glands of the insect, completing the life cycle^3,4^. Infection with *Leucocytozoon* species is most often subclinical, leading to economic losses due to suboptimal performance attributed to decreased feed intake, lower growth rate, lower body weight, and reduced production^5^. *L. caulleryi* is extremely pathogenic and causes fatal hemorrhagic disease in chickens. The disease is known as Bangkok hemorrhagic disease because it causes severe acute clinical symptoms such as anemia, lethargy, diarrhea, and large multifocal hemorrhages in numerous organs^6,7^. *L. caulleryi* can be diagnosed through microscopic examination of stained blood smears by detecting the distinctive gametocytes present in erythrocytes and leukocytes^3^. Histopathological examination is a crucial diagnostic step for identifying schizonts in various tissues^8^. Furthermore, the polymerase chain reaction (PCR) technique is used, which is more sensitive than microscopy, as it can detect the parasite’s DNA in thin blood smears even in the absence of gametocytes^9^. In Egypt, this avian outbreak first appeared in El-Beheira governorate in the summers of 2019 and 2020, infecting five broiler flocks with a mortality rate of 0.3-1%, accompanied by severe hemorrhagic PM findings^3^. In addition, a report documented a combined infection of *L. caulleryi* and *chicken anemia virus* in 25 broiler chicken farms and 3 broiler breeder farms across various governorates, resulting in a mortality rate of 3.2% to 9%, with extensive hemorrhages observed during PM examination^4^. More recently, *Leucocytozoon* infection in pigeons was recorded in Upper Egypt via the collection of blood samples from various local and household breeders, revealing a prevalence rate of 12.8%^10^. The rationale and hypothesis for the present study stem from the need to diagnose and understand the recently emerged avian outbreak in El-Sharkia governorate in Egypt, which appeared in 2023. This outbreak was characterized by extensive hemorrhages in the breast and thigh muscles, as well as multiple other organs during necropsy. These findings were initially confused with other diseases causing hemorrhagic syndrome in chickens, such as *infectious bursal disease (IBD)*,* chicken infectious anemia virus (CIAV)*,* Newcastle disease (ND*), and *Highly Pathogenic Avian Influenza (HPAI)*. However, these conditions can be differentiated from *L. caulleryi* infection by detecting distinctive gametocytes in blood smears and characteristic megaloschizonts in histopathological examinations of various organs, with confirmation through PCR analysis. This study reports the first detection of *L. caulleryi* infection in broiler chicken flocks in El-Sharkia governorate, Egypt, based on clinical, hematological, histopathological, and molecular investigations.

## Results

### Clinical examination and postmortem (PM) findings

**Fig. 1:**
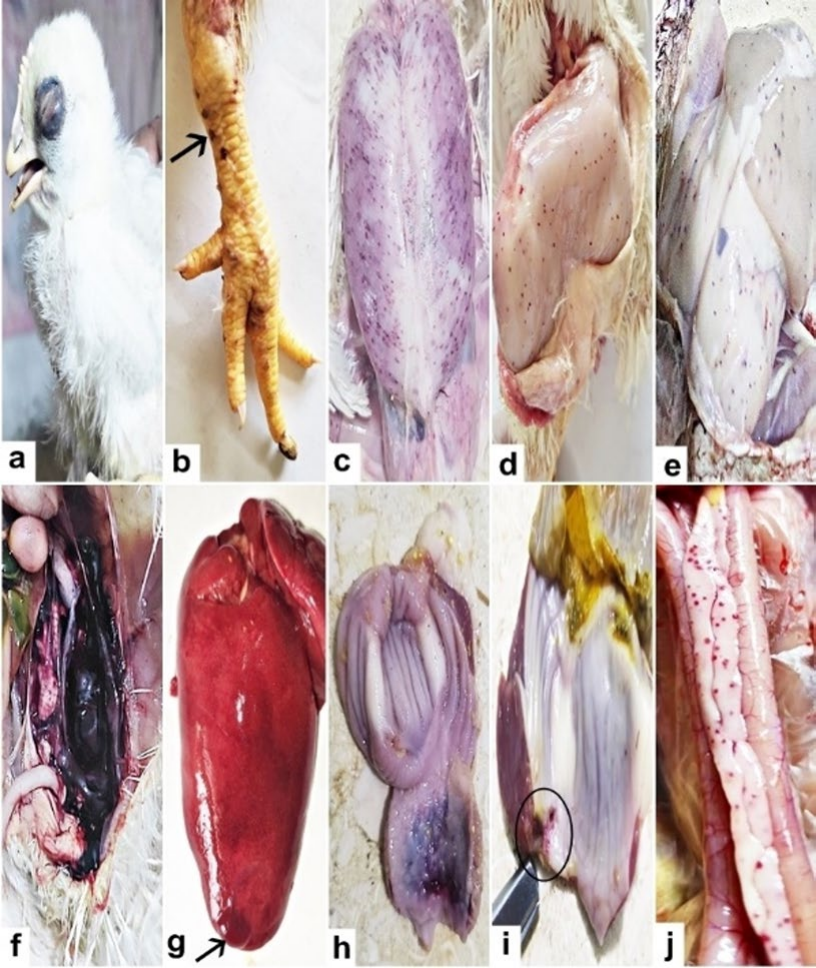
Gross lesions of naturally infected chicken with *L. caulleryi* collected at different time points and from different farms showed (**a**) subcutaneous hemorrhage and edema around the eyes, (**b**) multifocal to coalescing petechial subcutaneous rounded hemorrhagic nodules on the shanks and toes (arrow), (**c**–**e**) multifocal pin-headed hemorrhagic petechiae embedded in the breast, thigh muscles, and subcutaneous fat, (**f**) the kidneys were severely swollen and hemorrhagic, with a large pool of blood in the abdominal cavity, (**g**) hemorrhages under the capsule of the liver (arrow), (**h**) hemorrhagic patches present in the interior and exterior part of the proventriculus, (**i**) clear and obvious gizzard ulcer (circle) and (**j**) prominent petechial hemorrhages present in the pancreas were observed.

In this study, we focused on broiler poultry farms in El-Sharkia governorate. The affected chicken flocks were raised in open poultry farms located near agricultural rice fields, where biting *Culicoides* were observed inside the farms. The disease caused a mortality rate of 0.5% to 2% over a 7-day period, starting from the onset of noticeable clinical signs in the affected chickens. Clinical signs included varying degrees of lethargy, depression, loss of appetite, weight loss, ruffled feathers, pale combs and wattles, and subcutaneous hemorrhage and edema around the eyes (Fig. 1a). PM examination of the affected chickens revealed multifocal to coalescing petechial subcutaneous rounded hemorrhagic nodules on the shanks and toes (Fig. 1b) and multifocal pinhead-sized hemorrhagic petechiae embedded in the breast muscles (Fig. 1c), thigh muscles (Fig. 1d), and subcutaneous fat (Fig. 1e). The kidneys were severely swollen and hemorrhagic, with a large pool of blood in the abdominal cavity (Fig. 1f). Moreover, hemorrhages beneath the liver capsule (Fig. 1g), hemorrhagic patches on both the interior and exterior surfaces of the proventriculus (Fig. 1h), gizzard ulcers (Fig. 1i), and prominent petechial hemorrhages in the pancreas were observed (Fig. 1j).

### Hematological findings

**Fig. 2:**
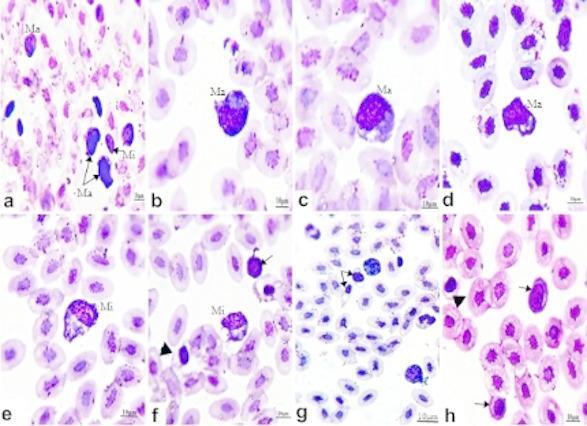
Giemsa-stained blood smears from naturally infected chickens showed various stages of *L. caulleryi* gametocytes replacing the host cell nucleus. (**a**) Macrogametocytes (Ma) exhibit a large deep basophilic color and microgametocyte (Mi) which is smaller in size with eosinophilic color. (**b**–**d**) Macrogametocytes (Ma) with large, deep basophilic color. (**e**) Microgametocyte (Mi) with eosinophilic color. (**f**) Microgametocyte (Mi) small in size with eosinophilic color, immature parasite stage, rounded in shape, and inducing a minimal effect on the morphology of the host cells (arrow) and deformity in the shape of red blood cells (arrowhead). (**g**) Deformity in the shape of red blood cells (arrows). (**h**) Immature parasite stage rounded in shape and induced a minimal effect on the morphology of the host cells (arrows) and deformities in the shape of red blood cells (arrowhead). Bare 10.

Microscopic examination of blood smears collected from infected chickens and stained with Giemsa revealed the presence of various developmental stages of *L. caulleryi*. The shape of gametocytes was oval or round, and they were found to undergo marked morphological changes in the host cells, which enlarged in size, and their nuclei became displaced from their normal sites (Fig. 2). The difference between male (microgametocytes) and female (macrogametocytes) in the Giemsa-stained blood film was achieved through distinct morphological and staining properties specific to each sex. Macrogametocytes showed a distinct deep basophilic color and were larger in size (Fig. 2a-d) than microgametocytes, which exhibited an eosinophilic or pinkish color (Fig. 2a, e, & f). Gametocytes induced deformity in the shape of red blood cells (Fig. 2f &g). In contrast, immature parasitic stages were rounded in shape, frequently observed in close proximity to the nucleus of the host cells, and had minimal impact on the morphology of the host cells (Fig. 2f & h).

### Cell block examination

**Fig. 3:**
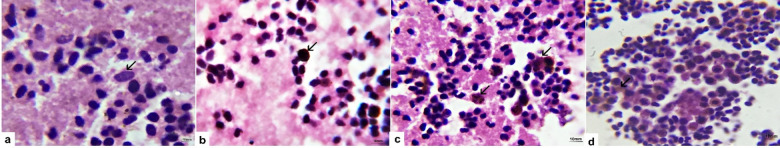
Cell block of blood paraffin sections from naturally infected chickens with *L. caulleryi*. H&E stain. (**a**) Mature stages of parasites free in the blood (arrow), (**b**) immature stages of parasite rounded in shape, and replacing the host cell nucleus without affecting morphology (arrow), and (**c**, **d**) hemolysis of many red blood cells with the presence of dark brownish hemosiderin pigments (arrows). Bare 10.

The examined cell blocks prepared from blood samples of naturally infected flocks revealed the presence of mature stages of the parasite free in the blood (Fig. 3a), immature stages of the parasite rounded in shape and replaced the host cell nucleus without altering its morphology (Fig. 3b), and hemolysis of many red blood cells accompanied by dark brown hemosiderin pigments (Fig. 3c & d).

### Histopathological examination

**Fig. 4:**
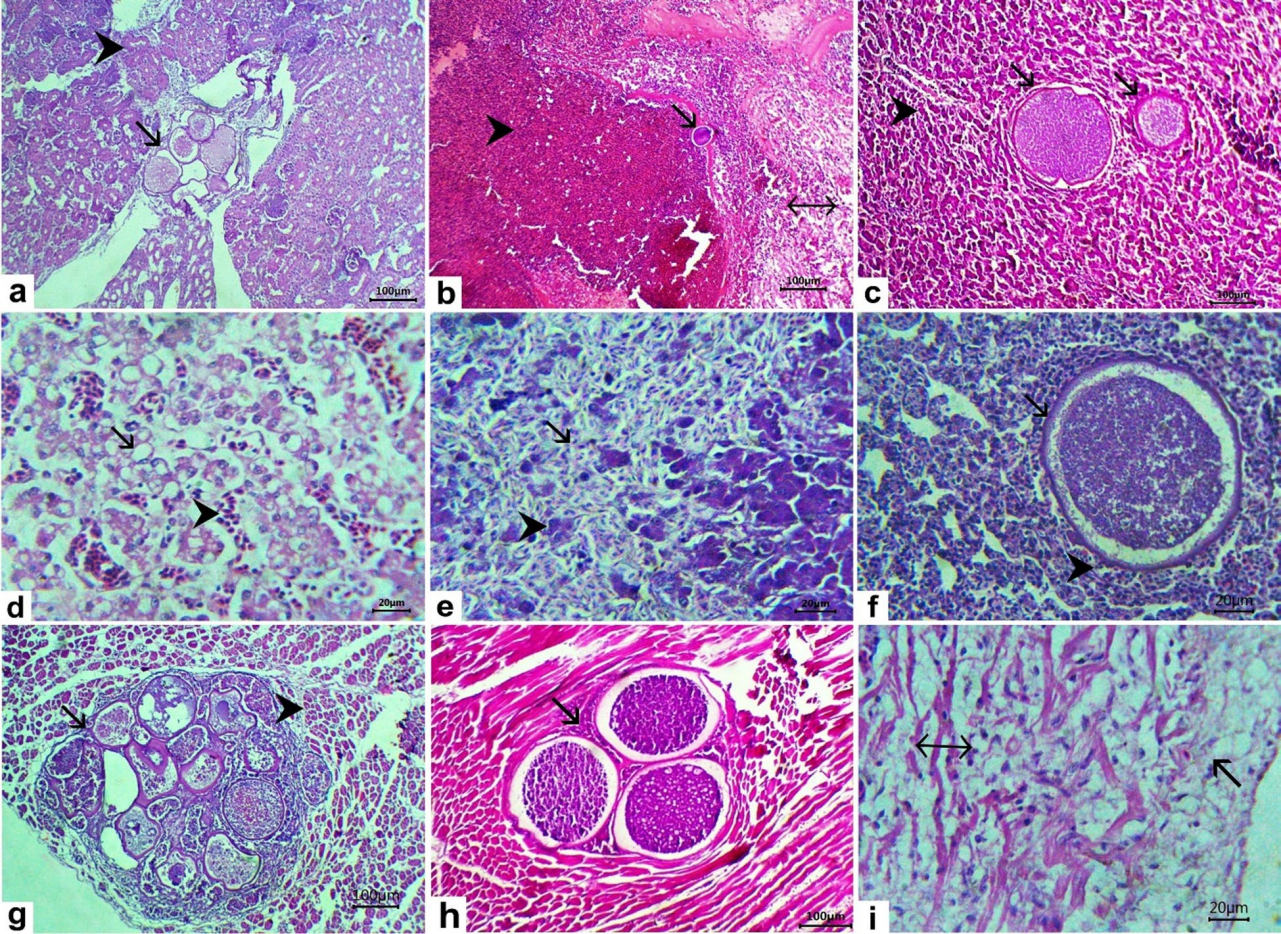
Histopathological lesions in tissues collected from chickens naturally infected with *L. caulleryi*. H&E stain. Kidney (**a**, **b**), (**a**) the kidney exhibited multiple irregular-shaped thick-walled megaloschizonts containing dark, round basophilic merozoites (arrow), the renal tubules showed cloudy swelling (arrowhead), bare 100. (**b**) An isolated megaloschizont was observed (arrow) with diffuse hemorrhage (arrowhead), and tubular necrosis and lymphocytic infiltration (double-headed arrow) bare 100. The liver (**c**–**e**): (**c**) the liver showed megaloschizonts containing numerous basophilic, round merozoites (arrows) with congestion of hepatic sinusoids (arrowhead) bare 100. (**d**) The hepatocytes showed vacuolation (arrow) and congestion of hepatic sinusoids (arrowhead) bare 20. (**e**) Necrosis of hepatocytes replaced by RBCs (arrow) and individualized hepatocytes (arrowhead) bare 20. (**f**) The lung showed megaloschizont with a thick wall containing numerous basophilic merozoites (arrow) and vascular congestion (arrowhead) bare 20. (**g**) The muscles showed multiple irregular-shaped thick-walled megaloschizonts replacing the muscle fibers (arrow) with hyalinization of muscles (arrowhead) bare 100. The heart (**h**, **i**): (**h**) showed multiple megaloschizonts replacing cardiac muscle (arrow) bare 100. (**i**) fibrinous pericarditis (arrow) and hyalinization of cardiac muscles with intermuscular edema (double-headed arrow) bare 20.

**Fig. 5:**
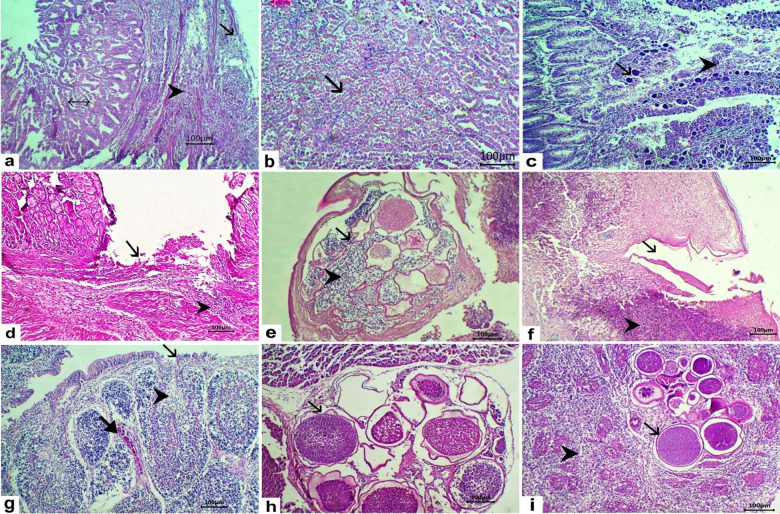
Histopathological lesions in tissues collected from chickens naturally infected with *L. caulleryi*. H&E stain. Proventriculus (**a**, **b**), (**a**) the mucosa showed erosion of the epithelium (arrow) and diffuse lymphocytic infiltration in the lamina propria (arrowhead), necrotic proventricular glands, and epithelial desquamation into the lumen (double-headed arrow) bare 100. (**b**) the proventricular glands showed necrosis and desquamation into the lumen (arrow) bare100. (**c**) The intestinal mucosa showed multiple megaloschizonts replacing the epithelium (arrow) with desquamated epithelium infiltrated by mononuclear cells mainly lymphocytes and macrophages (arrowhead) bare 100. (**d**) The gizzard showed an ulcer in the mucosa (arrow) with intermuscular infiltration of mononuclear cells (lymphocytes and macrophages) (arrowhead), bare 100. The skin (**e**, **f**), (**e**) the dermis showed multiple irregular-shaped thick-walled megaloschizonts (arrow), and numerous liberated merozoites were seen (arrowhead) bare 100. (**f**) An ulcer with exposure of the dermis (arrow) with a focal area of caseous necrosis in the dermis (arrowhead) bare 100. (**g**) The bursa showed erosion of epithelium (arrow) with necrosis and depletion of lymphocytes in the lymphoid follicle (arrowhead), and congestion of blood vessel (thick arrow) bare 100. (**h**) The pancreas showed multiple irregular-shaped thick-walled megaloschizonts containing dark, round merozoites (arrow) bare 100. (**i**) The spleen showed multiple irregular-shaped thick-walled megaloschizonts containing dark, round merozoites (arrow) with depletion of the white and red pulp (arrowhead) bare 100.

The kidneys exhibited multiple thick-walled, blood-filled megaloschizonts containing dark, round basophilic merozoites (Fig. 4a), accompanied by diffuse hemorrhage, tubular necrosis, and lymphocytic infiltration (Fig. 4b). The liver showed megaloschizonts containing numerous basophilic, round merozoites (Fig. 4c), congestion of hepatic sinusoids, and vacuolation (Fig. 4d), along with necrosis of hepatocytes replaced by RBCs and individualized hepatocytes (Fig. 4e). The lungs contained thick-walled megaloschizonts containing numerous merozoites and vascular congestion (Fig. 4f). The muscle tissue showed multiple irregularly shaped, thick-walled megaloschizonts that replaced the muscle fibers (Fig. 4g). The heart revealed multiple megaloschizonts replacing the cardiac muscle (Fig. 4h), fibrinous pericarditis, hyalinization of the cardiac muscle, and intermuscular edema (Fig. 4i). The proventricular mucosa showed erosion of the epithelium with diffuse lymphocytic infiltration in the lamina propria (Fig. 5a), necrosis of the proventricular gland, with epithelial desquamation into the lumen (Fig. 5b). The intestinal mucosa exhibited multiple megaloschizonts replacing the epithelium, with desquamated epithelium and infiltration by mononuclear cells mainly lymphocytes and macrophages (Fig. 5c). The gizzard showed an ulcer in the mucosa with intermuscular infiltration of mononuclear cells (lymphocytes and macrophages) (Fig. 5d). The dermal layer of the skin contained multiple irregularly shaped, thick-walled megaloschizonts containing numerous round merozoites (Fig. 5e). Ulcers with dermal exposure and focal areas of caseous necrosis were also observed (Fig. 5f). The bursa exhibited erosion of the epithelium, necrosis and depletion of lymphocytes in the lymphoid follicle, along with congestion of blood vessels (Fig. 5g). The pancreas contained multiple irregularly shaped thick-walled megaloschizonts containing dark, round merozoites (Fig. 5h). The spleen contained multiple thick-walled, irregularly shaped megaloschizonts containing dark, round merozoites and depletion of both white and red pulp (Fig. 5i).

### Real-time PCR detection

**Fig. 6:**
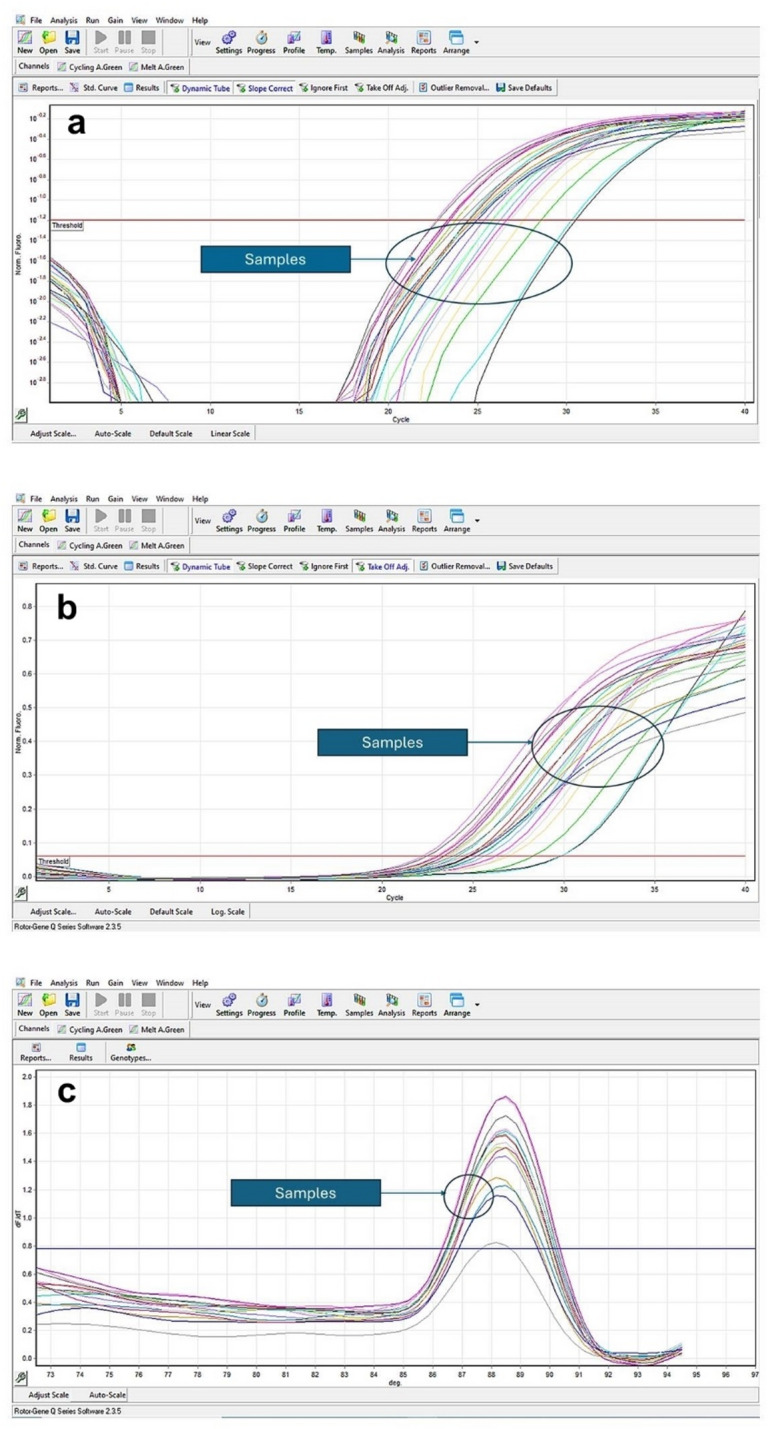
Real-time PCR analysis of samples collected from 24 broiler chicken flocks naturally infected with *L. caulleryi*. (**a**) Amplification blot log. (**b**) Amplification blot linear. (**c**) Melting curve analysis.

All examined samples collected from 24 broiler farms naturally infected with *L. caulleri* showed positive expression of *L. caulleri* (Fig. 6a), that was confirmed with the amplification blot curves (Fig. 6b) and melting curve analysis (Fig. 6c). CT values of real-time PCR for each farm are provided in Supplementary Data S1.

### Statistical analysis

#### Descriptive statistics (see Table 1)

**Table 1:** Descriptive statistics for age, weight, and mortality (*N* = 24). This table presents the minimum, maximum, mean, and standard deviation for three variables: age, weight, and mortality at the onset of clinical signs.

Variable	*N*	Minimum	Maximum	Mean	Std. deviation
Age (days)	24	10.00	35.00	24.17	6.85
Weight (g)	24	284.00	2230.00	1268.29	559.90
Mortality (%)	24	0.50	2.00	1.14	0.39

#### Normality test

The Shapiro-Wilk test for mortality rate yielded non-significant results (p-values: 0.391 and 0.885), indicating that the data were normally distributed (assumption of t-test).

#### Independent t-test (see Tables 2 and 3)

**Table 2:** Comparison of mortality rates between 2023 and 2024. The difference in mortality rates between 2023 and 2024 was not significant.

Year	*N*	Mortality Mean ± SEM	Sig. (2-tailed)	Mean difference	Std. error difference	Comment
2023	9	1.033 ± 0.11	0.296^Ns^	− 0.17333	0.16204	Non significant difference across years
2024	15	1.206 ± 0.11				

Ns: Non-significant at *p* > 0.05.

**Table 3:** Pearson correlation between age and mortality. The pearson correlation coefficient (*r* = − 0.836), a negative correlation, indicates a very strong inverse linear relationship between age and mortality percentage. As age increases, the mortality percentage tends to decrease.

Mortality	
Age	− 0.836**
Sig. (2-tailed)	0.000**

**Significance at *p* < 0.01.

### Post-hoc power analysis

With 9 flocks in 2023 and 15 flocks in 2024, post-hoc analysis suggested a statistical power of (~ 20%) and Cohen’s d (≈ 0.45). While preliminary results showed no statistically significant difference in mortality percentage, the post-hoc power analysis indicated that the sample size was underpowered to detect small differences.

## Discussion

The impetus for this study was the need to understand the recently emerged leucocytozoonosis in Egypt, a disease often confused with other conditions causing hemorrhagic syndrome in chickens. From this perspective, it became imperative to thoroughly examine numerous farms exhibiting clinical signs using well-established diagnostic methods, while also incorporating innovative diagnostic approaches. This study is the first to detect *L. caulleryi* infection in broiler chicken flocks in El-Sharkia governorate, Egypt. In Egypt, excessive humidity and rising temperatures, combined with inadequate biosecurity practices in chicken farms, create an ideal environment for biting midges reproduction^4^. Therefore, most of the examined broiler chicken farms were reared in open farms and located close to water sources and agricultural areas, which are geographically associated with the habitat of the intermediate host. The mortality rates reported in this study were relatively low, varying between 0.5% and 2%, which was relatively consistent with the clinical impact observed in the *L. caulleryi* outbreak in commercial layer flocks, which exhibited mortality rates between 0.6 and 2.3%^11^ and were slightly higher than those reported for *L. caulleryi* infection in commercial broilers in Egypt, with mortality rates ranged from 0.3 to 1%^3^. The variation in the percentage of mortality rates and disease pathogenicity are multifactorial, depending on several factors, including the strain of the parasite, the initial inoculum of sporozoites, the presence of single or mixed parasitic infections, the avian species affected, the degree of exposure, and the age and immunological status of the host, as well as other contributing factors^3^. The Shapiro-Wilk test confirmed that the data were normally distributed, allowing for the use of parametric tests to compare mortality rates across years. Changes in vector habitats and their distribution caused by climate change and global warming^11^ contributed to the non-significant increase in mortality between 2023 and 2024. A key finding was a strong negative correlation between age and mortality, indicating that younger birds had higher mortality rates. This finding was in agreement with^12^, who documented that young birds are most susceptible to leucocytozoonosis and may die within a short time after infection due to immature immune responses and underdeveloped physiological defenses. Unfortunately, the post hoc power analysis revealed low statistical power, suggesting that the sample size was insufficient. This limitation is largely attributable to the recent emergence of the disease, first observed locally in the summer of 2023, which appears to exhibit a strong seasonal pattern only during the summer months. Consequently, opportunities to collect data across multiple flocks or time points were inherently limited during the early phase of the outbreak. The clinical signs observed in this study were similar to those reported by^3^, who described that the signs of *L. caulleryi* infection in broilers were lethargy, depression, loss of appetite, weight loss, ruffled feathers, and pale combs and wattles. *L. caulleryi* infection in domestic chickens is known as Bangkok hemorrhagic disease in Asia^12^, which correlates with our postmortem findings represented by widespread hemorrhages across multiple organs. The clinical manifestations and mortality associated with this disease are primarily attributed to intravascular hemolytic anemia induced by anti-erythrocytic factors^13^. The hypothesis that intravascular hemolytic anemia results from the mechanical destruction of RBCs by gametocytes was excluded and instead relied upon the principle that, as the lowest hematocrit values occurred prior to the peak of parasitemia, the anemia is likely associated with an anti-erythrocyte factor, which is released either by the merozoites or their host cells^14^. Moreover, the mortalities were also caused by severe pain, tissue damage, and hemorrhage in the peritoneal cavity, kidneys, and subdural cavity that occurred during the release of second-generation merozoites from the megaloschizonts^4^. The Giemsa-stained blood smears in our study were similar to those reported by^3,15^, who identified the presence of microgametocytes and macrogametocytes and reported that the differences between them were achieved through distinct morphological and staining characteristics specific to each sex, as macrogametocytes exhibited a deep basophilic color and were larger, whereas microgametocytes displayed an eosinophilic or pinkish color. Furthermore, the study of^3^ reported that mature gametocytes induced significant morphological changes in host cells, which enlarged and exhibited displaced nuclei, while immature parasite stages had a minimal impact on the morphology of the host cells, which was similar to our observations. Electron microscopy clarified the differences in staining characteristics between macrogametocytes and microgametocytes. Macrogametocytes have been observed to contain more densely packed ribosomal content than microgametocytes, and the ribosomes have a large content of ribonucleic acid, which takes the basophilic color^16^. It’s remarkable that *L. caulleryi* gametocytes lack pigment granules, a key feature that distinguishes them from avian malaria gametocytes. Furthermore, erythrocytes infected with *L. caulleryi* didn’t display intra-erythrocytic schizonts, a characteristic feature observed in avian malaria infections^16^. These findings provide clear diagnostic criteria for differentiating *L. caulleryi* from avian malaria parasites. Here, we first introduce the cell block technique for the examination of *L. caulleryi* as a reliable diagnostic tool, particularly in cases when blood smears are inadequate or inconclusive. Also, the cell block allows the examination of a larger volume of blood samples than that used for blood smears, increasing the likelihood of diagnosing early or late infections when the parasite is scanty in the blood. The cell block technique maintains cells and tissue architecture, facilitating a detailed examination of the morphology of the parasite within host cells^17^, which aligns precisely with our findings. Furthermore, the cell block enables in the preparation of multiple sections from the same sample, allowing for various staining methods with different stains, thereby improving the detection of parasites present in low numbers^18^. At the histopathological level, which is considered the definitive diagnostic method for avian leucocytozoonosis, studies of^3,12,19^ reported the presence of numerous characteristic thick-walled megaloschizonts containing many basophilic, round merozoites in multiple organs, including the kidneys, liver, lungs, muscles, heart, intestine, pancreas, and spleen, beside hemorrhages, necrosis, with a significant lymphocytic infiltration, which was clearly similar to the findings of this study. Moreover, our study identified an ulcer in the gizzard mucosa with intermuscular infiltration of mononuclear cells. This ulcer likely resulted from severe anemia, which leads to tissue ischemia and hypoxia, weakening the gizzard mucosa and facilitating ulcer formation. We believe this aspect needs further investigation, considering the possibility that the parasite has recently altered its pathological behavior. The study by^4^ reported caseous necrosis in the dermis of the legs, which partially aligns with our findings. Real-time PCR is highly sensitive and reliable for detecting the presence of *L. caulleri* in tissue samples. The primers described by^20^ effectively amplify *L. caulleri* DNA using real-time PCR. Our results showed that all samples collected from the 24 farms showed a positive sense for *L. caulleri*. The CT values for the examined samples appeared early at 20–35 cycles, indicating a high abundance of *L. caulleri* in the examined tissue. This was further confirmed by the amplification blot curve and melting curve analysis. There are some limitations of the present study, such as a small sample size, which is due to the recent outbreak in El-Sharkia governorate. This outbreak occurred only during the summer season (from August’s second half to early October) and is associated with the presence of vectors in open poultry farms.

In conclusion, this study represents the first confirmed detection of *L. caulleri* infection in broiler chicken flocks in El-Sharkia governorate, Egypt, during the late summer seasons of 2023 and 2024. This study provides key clinical, hematological, histopathological, and molecular analyses of the disease, facilitating accurate diagnosis and differentiation of *L. caulleri* infection from other hemorrhagic diseases in poultry. The implications of this study are important for the poultry industry, achieved through increasing awareness and knowledge among veterinarians and poultry producers about this occult disease and highlighting the need for improved biosecurity. Future perspectives research should focus on conducting large-scale surveillance to monitor the spread of *L. caulleryi* across poultry farms in El-Sharkia and other Egyptian governorates. This will enhance understanding and provide comprehensive data on its prevalence and epidemiology. Furthermore, to mitigate its impact on poultry production, it is essential to develop effective preventive and therapeutic interventions together with strengthened biosecurity strategies.

## Materials and methods

### Study design & chickens history

This study was conducted in El-Sharkia governorate, located in northeastern Egypt (30°35′02″ N, 31°31′13″ E), during the summers of 2023 and 2024. Initially, we examined 32 affected broiler chicken flocks comprising a total of 276,000 birds from different areas within El-Sharkia governorate. Out of them, 24 affected broiler chicken flocks with a total number of 180,000 birds, were included in the study based on specific inclusion criteria: affected chickens reared in open poultry farms near water sources and rice cultivation areas; clinical signs including decreased feed intake, pale comb and wattle; low mortality rates (less than 2%); hemorrhagic syndrome observed during post-mortem examination; no history of treatment with sulfonamide, and vaccinated against variable viral diseases according to the age of each flock. The selected birds for examination included an equal distribution of males and females, with varying ages and weights recorded at diagnosis, and mortality rates ranged from 0.5% to 2% within 7 days starting from the onset of clinical signs (Supplementary Table S1). Flocks were excluded if they had a history of previous infections, exhibited high mortality rates (exceeding 20%), or were reared in closed poultry systems or desert environments lacking vector habitats.

### Ethical approval

In the present study, we exert all efforts to minimize the suffering of birds. All procedures, including samples collection, all examinations, and tests, were performed in accordance with the Ethical Norms approved by the Institutional Animal Care and Use Committee (IACUC) of Zagazig University (ZU-IACUC committee approval number ZU-IACUC/2/F/269/2024). Our study was conducted in accordance with ARRIVE guidelines. Consents were obtained from all farm owners in El-Sharkia governorate prior to sampling. The purpose of the study was explained, and all farm owners agreed to the use of data and findings for research and publication purposes.

### Clinical, PM procedures, and samples collection

The clinical investigation of suspected leucocytozoonosis in chickens began with the initial identification of cases by monitoring the flock for early clinical signs such as anorexia, weakness, ruffled feathers, pale comb and wattles (anemia), and mortalities. Affected birds were subsequently isolated for thorough physical examination. Additionally, environmental conditions were assessed, revealing a high population of *Culicoides* midges within the poultry houses, suggesting the presence of a potential vector. PM examinations were conducted by collecting recently dead birds, along with five diseased birds randomly selected from each flock. These birds were humanely euthanized using intravenous sodium pentobarbital (50 mg/kg) to record the PM findings^3^. External examination focused on pallor of the comb, wattles, and mucous membranes, as well as the presence of hemorrhagic nodules on the shanks and toes. Necropsy procedures included examination of the muscles, subcutaneous fat, liver, proventriculus, gizzard, intestine, pancreas, kidneys, lungs, heart, spleen, and bursa of Fabricius. To minimize potential confounding factors, consistent sampling procedures were implemented across all flocks, including standardized criteria for sample collection and handling. Additionally, data were collected from multiple geographic locations and flock types to reduce location-specific or management-related biases.

### Hematological examination

Blood samples were collected from five diseased birds per flock. First, the brachial area was disinfected with 70% ethanol. Then, using a fresh syringe and needle, blood samples (2 ml/bird) were drawn from the brachial wing vein, and the blood was immediately transferred to 2 ml EDTA tubes and mixed thoroughly with anticoagulant. Samples were kept in an icebox until processing. A thin blood smear was prepared on the same day of blood collection^7^. A drop of blood (10–20 µl) was spread onto a clean slide, then completely air-dried for 5–10 s, fixed with methanol for 5 min, and stained with 10% Giemsa solution for 15 min^21^. Blood films were examined for the presence of gametocytes under the oil immersion lens of a light microscope. Images were captured by a Leica microscope provided with a camera (Leica Microsystems Inc., Buffalo Grove, IL).

### Cell block examination

The cell block is a sample processing technique in which a routine cytology sample is concentrated and then processed as a histopathological sample with the basic steps of cellular concentration, sample fixation, and paraffin embedding. Briefly, the blood sample was mixed with formalin in a 10 mL centrifuge tube and centrifuged at 1000 rpm for 10 min. The supernatant was decanted, and 70% ethyl alcohol was added to the sediment. The sediment was resuspended by agitation and centrifuged again at 1000 rpm for 10 min. The sediment was then wrapped in lens paper, placed in a tissue cassette, processed in xylene, embedded in paraffin^18^, sectioned at 5 μm thickness, and examined microscopically after staining with hematoxylin and eosin^22^. Images were captured using a Leica microscope provided with a camera (Leica Microsystems Inc., Buffalo Grove, IL).

### Histopathological examination

Specimens from affected tissues including breast and thigh muscles, kidneys, liver, proventriculus, gizzard, intestine, spleen, heart, lungs, skin of the shank, and bursa of Fabricius were collected from both diseased as well as recently dead chickens. The tissue specimens were fixed in 10% neutral buffered formalin for 72 h. Post fixation, the tissue specimens were washed in tap water, dehydrated in ascending grades of ethyl alcohol, cleared in Histo-Choice^®^ (Sigma-Aldrich, St. Louis, USA) clearing agent, impregnated, and embedded in paraffin wax, sectioned at 5 μm thickness, stained with hematoxylin and eosin stain, and examined microscopically^22^. Images were taken by a Leica microscope provided with a camera (Leica Microsystems Inc., Buffalo Grove, IL).

### Real-time PCR detection

Tissue samples from five diseased chickens in each flock were collected and pooled as one tissue homogenate per flock. DNA was extracted using the Qiagen DNeasy Tissue Kit (Qiagen, Germany), following the manufacturer’s instructions for DNA extraction from tissue. All reactions were performed on a real-time thermocycler (Rotor-Gene Q 2plex) using iTaq Universal SYBR Green Supermix. The extracted nucleic acids were subjected to molecular identification using a specific pair of primers for *L. caulleryi* L545F: (5’ -ACAAATGAGTTTCTGGGGA-3’) and L825R: (5’ -GCAATTCCAAATAAACTTTGAA- 3’) to amplify the mitochondrial cytochrome oxidase b gene of *L. caulleryi* DNA. The reaction volume was 15 µl, containing 7.5 µl of SYBR Green Supermix, 0.6 µl of each primer (10 µM concentration), 3.3 µl of molecular-grade water, and 3 µl of DNA template (estimated volume at 20 ng/ µl). The cycling conditions were as follows: 95 °C for 30 s, followed by 35 cycles of 95 °C for 30 s and 53 °C for 35 s (with plate reading), and finally a melt curve analysis using the instrument’s default parameters^20^.

### Statistical analysis

Descriptive statistics (Table 1) are presented as mean ± standard error of the mean (SEM). The normality of the data was assessed using the Shapiro-Wilk test. Independent t-tests were used to compare mortality rates across different years (Table 2). Pearson correlation analysis was conducted to examine the relationship between age and mortality (Table 3). A p-value less than 0.05 was considered statistically significant. Post hoc power analysis was performed using G*Power 3.1.9.7 for independent samples t-test. A post hoc power analysis was conducted using the means and standard errors of the two groups. The first group (2023) had a mean mortality rate of 1.033% (SE = 0.1067), while the second group (2024) had a mean of 1.206% (SE = 0.1075). The effect size (Cohen’s d) was calculated to be approximately 0.45, indicating a small to moderate effect. Using an independent two-sample t-test with unequal group sizes (n_1_ = 9, n_2_ = 15) a significance level of 0.05 (two-tailed), achieved statistical power was found to be approximately 20%. All statistical analyses and graphical illustrations were performed using IBM SPSS Statistics for Windows, Version 24.0 (IBM Corp., Armonk, NY, USA)^23^.

## Supplementary Information

Below is the link to the electronic supplementary material.

Supplementary Material 1

Supplementary Material 2

## Data Availability

The datasets used and analyzed in this study are available from the corresponding authors upon reasonable request.
