# Supplementary material for: New insights into recently emerged Leucocytozoon caulleri infection in Egyptian broiler flocks through clinical, pathological, hematological, and molecular investigation
Source: Sci Rep. 2025 Dec 3;15:43091. doi: 10.1038/s41598-025-26311-7 (PMC12675588; doi:10.1038/s41598-025-26311-7)
Supplement: Supplementary file 2 — Supplementary Material 2 [file 41598_2025_26311_MOESM2_ESM.pdf]

Supplementary Material

New insights into recently emerged *Leucocytozoon caulleri* infection in Egyptian broiler flocks through clinical, pathological, hematological, and molecular investigation

Moustafa S. Abou El-Fetouh [1], Nahla AG. Ahmed Refat [1], Nora M. Elseddawy [1], Tarek Khamis [2]* & Mohamed A.A. Abdalla [1]*

[1] Department of Pathology, Faculty of Veterinary Medicine, Zagazig University, Zagazig 44519, Egypt.
[2] Department of Pharmacology, Faculty of Veterinary Medicine, Zagazig University, Zagazig 44519, Egypt.
[2] Laboratory of Biotechnology, Faculty of Veterinary Medicine, Zagazig University, Zagazig 44519, Egypt.

Supplementary Table S1: History of the 24 examined broiler flocks from El-Sharkia governorate, Egypt.

Flock	Number	Age (days)	Average body weight (gm)	Year	Mortality (%)
**1**	5000	15	532	2023	1.5
**2**	3500	22	1030	2023	1
**3**	8000	32	1945	2023	0.6
**4**	12000	25	1292	2023	0.8
**5**	6000	18	736	2023	1.5
**6**	22000	20	880	2023	1.2
**7**	16000	25	1285	2023	1
**8**	4000	32	1950	2023	0.7
**9**	7500	28	1560	2023	1
**10**	5000	25	1290	2024	1.2
**11**	9500	16	600	2024	1.6
**12**	4500	25	1287	2024	0.8
**13**	3000	17	668	2024	1.5
**14**	7500	20	880	2024	1.4
**15**	10000	33	2045	2024	0.8
**16**	5500	27	1475	2024	1
**17**	3000	10	284	2024	2
**18**	3500	33	2052	2024	0.5
**19**	7000	26	1386	2024	1.4
**20**	15000	35	2230	2024	1.3

**21**	5000	13	425	2024	1.8
**22**	6500	28	1567	2024	1
**23**	3000	25	1280	2024	1
**24**	8000	30	1760	2024	0.8
